# Multiple Subject Barycentric Discriminant Analysis (MUSUBADA): How to Assign Scans to Categories without Using Spatial Normalization

**DOI:** 10.1155/2012/634165

**Published:** 2012-04-05

**Authors:** Hervé Abdi, Lynne J. Williams, Andrew C. Connolly, M. Ida Gobbini, Joseph P. Dunlop, James V. Haxby

**Affiliations:** ^1^School of Behavioral and Brain Sciences, University of Texas at Dallas, MS: GR4.1, 800 West Campbell Road, Richardson, TX 75080-3021, USA; ^2^The Rotman Institute at Baycrest, 3560 Bathurst Street, Toronto, ON, Canada M6A 2E1; ^3^Psychological Brain Sciences, Dartmouth College, 6207 Moore Hall, Hanover, NH 03755, USA; ^4^Dipartimento di Psicologia, Universitá di Bologna, Viale Berti Pichat 5, 40127 Bologna, Italy

## Abstract

We present a new discriminant analysis (DA) method called Multiple Subject Barycentric Discriminant Analysis (MUSUBADA) suited for analyzing fMRI data because it handles datasets with multiple participants that each provides different number of variables (i.e., voxels) that are themselves grouped into regions of interest (ROIs). Like DA, MUSUBADA (1) assigns observations to predefined categories, (2) gives factorial maps displaying observations and categories, and (3) optimally assigns observations to categories. MUSUBADA handles cases with more variables than observations and can project portions of the data table (e.g., subtables, which can represent participants or ROIs) on the factorial maps. Therefore MUSUBADA can analyze datasets with different voxel numbers per participant and, so does not require spatial normalization. MUSUBADA statistical inferences are implemented with cross-validation techniques (e.g., jackknife and bootstrap), its performance is evaluated with confusion matrices (for fixed and random models) and represented with prediction, tolerance, and confidence intervals. We present an example where we predict the image categories (houses, shoes, chairs, and human, monkey, dog, faces,) of images watched by participants whose brains were scanned. This example corresponds to a DA question in which the data table is made of subtables (one per subject) and with more variables than observations.

## 1. Introduction

A standard problem in neuroimaging is to predict category membership from a scan. Called “brain reading” by Cox and Savoy [1], and more generally multi-voxel pattern analysis (MVPA, for a comprehensive review see, e.g., [2]), this approach is used when we want to “guess” the type of category of stimuli processed when a participant was scanned and when we want to find the similarity structure of these stimulus categories (for a review, see, e.g., [3]). For datasets with the appropriate structure, this type of problem is addressed in multivariate analysis with discriminant analysis (DA). However, the structure of neuroimaging data precludes, in general, the use of DA. First, neuroimaging data often comprise more variables (e.g., voxels) than observations (e.g., scans). In addition, in the MVPA framework (see, e.g., the collection of articles reported in [2]), *f* mri data are collected as multiple scans per category of stimuli and the goal is to assign a particular scan to its category. These *f* mri data do not easily fit into the standard framework of DA, because DA assumes that one row is one observation (e.g., a scan or a participant) and one column is a variable (e.g., voxel). This corresponds to designs in which one participant is scanned multiple times or multiple participants are scanned once (assuming that the data are spatially normalized—e.g., put in Talairach space). These designs can fit PET or SPECT experiments but do not fit standard *f* mri experiments where, typically, multiple participants are scanned multiple times. In particular, DA cannot accommodate datasets with different numbers of variables per participant (a case that occurs when we do not use spatial normalization). Finally, statistical inference procedures of DA are limited by unrealistic assumptions, such as normality and homogeneity of variance and covariance.

In this paper, we present a new discriminant analysis method called Multiple Subject Barycentric Discriminant Analysis (MUSUBADA) which implements a DA-like approach suitable for neuroimaging data. Like standard discriminant analysis, MUSUBADA is used to assign observations to predefined categories and gives factorial maps in which observations and categories are represented as points, with observations being assigned to the closest category. But, unlike DA, MUSUBADA can handle datasets with multiple participants when each participant provides a different number of variables (e.g., voxels). Each participant is considered as a subtable of the whole data table and the data of one participant can also be further subdivided into more subtables which can constitute, for example, regions of interest (ROIs). MUSUBADA processes these subtables by projecting portions of the subtables on the factorial maps. Consequently, MUSUBADA does not require spatial normalization in order to handle “group analysis.” In addition, MUSUBADA can handle datasets with a number of variables (i.e., voxels) larger than the number of observations.

We illustrate MUSUBADA with an example in which we predict the type of images that participants' were watching when they were scanned. For each participant, one *anatomical* ROI was used in the analysis. Because the ROIs were anatomically defined, the brain scans were *not* spatially normalized and the corresponding number of voxels, as well as their locations, were different for each participant.

We decided to use hand-drawn ROIs,we could also have use functional localizers as these are still widely used (and constitute a valid approach as long as the localizer is not confounded with the experimental tasks). Hand drawing these ROIs is obviously very time consuming and could restrict the use of our technique to only small *N* studies (or to very dedicated teams) and therefore could also make studies harder to replicate. Fortunately, there are ways of obtaining ROIs without drawing them by hand. Specifically, as an alternative to manual tracing and functional localizers, recent methods have been developed to choose ROIs for each subject which are both automatic and *a priori*. These methods take labels from a standard anatomical atlas such as AAL [4], Tailarach [5], or Brodman [6] and warp these labels to coordinates within each subject's anatomical space. The anatomical coordinates are then downsampled to the subject's functional space. These steps can either be completely automated via extensions to standard software, such as IBASPM [7] or by reusing built-in tools, such as the linear FLIRT [8] and nonlinear FNIRT [9] of FSL. Because standard single-subject atlases do not account for between-subject variation [10], it may be preferable to use probabilistic atlases determined on multiple subjects (e.g., [11]). As an alternative to anatomical atlases entirely, stereotactic coordinates can also be taken from a meta-analysis and warped into coordinates within the subject's functional space. Although meta-analyses are generally performed for the task at hand, methods exist for automating even meta-analyses using keywords in published articles (see, e.g., NeuroSynth: [12]).

### 1.1. Overview of the Method

 MUSUBADA comprises two steps: (1) barycentric discriminant analysis (BADA) analyzes a data table in which observations (i.e., scans) are rows and in which variables (i.e., voxels) are columns and where each participant is represented by a subset of the voxels (i.e., one participant is a “subtable” of the whole data table), (2) and projection of the subtables representing the participants on the solution computed by BADA (this is the “MUSU” step in MUSUBADA). In addition, the subtable representing one participant could also be further subdivided into subtables representing, for example, the participant's ROIs (the ROIs can differ with the participants).

 BADA generalizes discriminant analysis and, like DA, it is performed when measurements made on some observations are combined to assign observations to *a-priori* defined categories. BADA is, actually, a class of methods which all rely on the same principle: each category of interest is represented by the *barycenter* of its observations (i.e., the weighted average; the barycenter is also called the *center of gravity* of the observations of a given category), and, then, a generalized principal component analysis (GPCA) is performed on the category by variable matrix. This analysis gives a set of discriminant factor scores for the categories and another set of factor scores for the variables. The original observations are then projected onto the category factor space, providing a set of factor scores for the observations. The distance of each observation to the set of categories is computed from the factor scores and each observation is assigned to the closest category.

The comparison between the *a-priori* and *a-posteriori* category assignments is used to assess the quality of the discriminant procedure. When the quality of the model is evaluated for the observations used to build the model, we have a *fixed* effect model. When we want to estimate the performance of the model for *new* or future observations, we have a *random* effect model. In order to estimate the quality of the random effect model, the analysis is performed on a subset of the observations called the *training* set and the predictive performance is evaluated with a different set of observations called the *testing* set. A specific case of this approach is the “leave-one-out” technique (also called jackknife) in which each observation is used, in turn, as the testing set whereas the rest of the observations play the role of the training set. This scheme has the advantage of providing an approximately unbiased estimate of the generalization performance of the model [13]. The quality of the discrimination can also be evaluated with an *R* ^2^-type statistic which expresses the proportion of the data variance explained by the model. Its significance can be evaluated with standard permutation tests.

The stability of the discriminant model can be assessed by a resampling strategy such as the bootstrap (see [13, 14]). In this procedure, multiple sets of observations are generated by sampling with replacement from the original set of observations, and by computing new category barycenters, which are then projected onto the original discriminant factor scores. For convenience, the confidence intervals of the barycenters can be represented graphically as a *confidence* ellipsoid that encompasses a given proportion (say 95%) of the barycenters. When two category ellipsoids do not intersect, these groups are significantly different.

**Figure 1:**
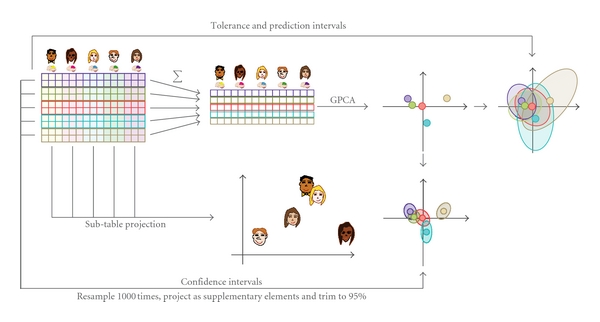
The different steps of BADA.

The problem of multiple tables corresponds to MUSUBADA  *per se* and it is implemented after the BADA step. In the MUSUBADA step, each subtable is projected onto the factor scores computed for the whole data table. These projections are also barycentric as their average gives the factor scores of the whole table. This last step integrates other multitable techniques such as multiple factor analysis or STATIS [15–19] which have also been used in brain imaging (see, e.g., for recent examples [20–22]). In addition to providing subtable factor scores, MUSUBADA evaluates and represents graphically the importance (often called the *contribution*) of each subtable to the overall discrimination. A sketch of the main steps of MUSUBADA is shown in Figure 1.

 MUSUBADA incorporates BADA which, itself, is a GPCA performed on the category barycenters and as such MUSUBADA implements a discriminant analysis version of different multivariate techniques such as correspondence analysis, biplot analysis, Hellinger distance analysis, and canonical variate analysis (see, e.g., [23–26]). In fact, for each specific type of GPCA, there is a corresponding version of BADA. For example, when the GPCA is correspondence analysis, this gives the most well-known version of BADA: discriminant correspondence analysis (DICA, sometimes also called correspondence discriminant analysis; see [23, 27–31]).

## 2. Notations

Matrices are denoted with bold uppercase letters (i.e., **X**) with generic element denoted with the corresponding lowercase italic letter (i.e., *x*). The identity matrix is denoted **I**. Vectors are denoted with bold lowercase letter (i.e., **b**) with generic element denoted with the corresponding lower case italic (i.e., *b*).

The original data matrix is an *N* observation by *J* variables matrix denoted **X**. Prior to the analysis, the matrix **X** can be preprocessed by centering (i.e., subtracting the column mean from each column), by transforming each column into a *Z*-score, or by normalizing each row so that the sum of its elements or the sum of its squared elements is equal to one (the rationale behind these different types of normalization is discussed later on). The observations in **X** are partitioned into *I* *a-priori* categories of interest with *N*_*i*_ being the number of observations of the *i*th category (and so ∑_*i*_^*I*^ *N*_*i*_ = *N*). The columns of matrix **X** can be arranged in *K* *a priori* subtables. The numbers of columns of the *k*th subtable are denoted *J*_*k*_ (and so ∑_*k*_^*K*^ *J*_*k*_ = *J*). So, the matrix **X** can be decomposed into *I* by *K* blocks as

(1) $$\begin{array}{c} X=\begin{array}{cc} & \begin{array}{ccccc} 1\; & \cdots \; & k\; & \cdots \; & K \end{array}\\ \begin{array}{c} 1\\ \vdots \\ i\\ \vdots \\ I \end{array} & \left[\begin{array}{ccccc} X_{1,1} & \cdots & X_{1,k} & \cdots & X_{1,K}\\ \vdots & \ddots & \vdots & \ddots & \vdots \\ X_{i,1} & \cdots & X_{i,k} & \cdots & X_{i,K}\\ \vdots & \ddots & \vdots & \ddots & \vdots \\ X_{I,1} & \cdots & X_{I,k} & \cdots & X_{I,K} \end{array}\right] \end{array}. \end{array}$$

### 2.1. Notations for the Categories (Rows)

We denote by **Y** the *N* by *I* design (aka dummy) matrix for the categories describing the rows of **X**: *y*_*n*,*i*_ = 1 when row *n* belongs to category and *i*, *y*_*n*,*i*_ = 0, otherwise. We denote by **m** the *N* by 1 vector of *masses* for the rows of **X** and by **M** the *N* by *N* diagonal matrix whose diagonal elements are the elements of **m** (i.e., using the diag operator which transforms a vector into a diagonal matrix, we have **M** = diag⁡{**m**}). Masses are positive numbers and it is convenient (but not necessary) to have the sum of the masses equal to one. The default value for the mass of each observation is often 1/*N*. We denote by **b** the *I* by 1 vector of masses for the categories describing the rows of **X** and by **B** the *I* by *I* diagonal matrix whose diagonal elements are the elements of **b**.

### 2.2. Notations for the Subtables (Columns)

We denote by **Z** the *J* by *K* design matrix for the subtables from the columns of **X**: *z*_*j*,*k*_ = 1 if column *j* belongs to subtable *k*, *z*_*j*,*k*_ = 0, otherwise. We denote by **w** the *J* by 1 vector of *weights* for the columns of **X** and by **W** the *J* by *J* diagonal matrix whose diagonal elements are the elements of **w**. We denote by **c** the *K* by 1 vector of weights for the subtables of **X** and by **C** the *K* by *K* diagonal matrix whose diagonal elements are the elements of **c**. The default value for the weight of each variable is 1/*J*, a more general case requires only **W** to be positive definite and this includes nondiagonal weight matrices.

## 3. Barycentric Discriminant Analysis (BADA)

The first step of BADA is to compute the barycenter of each of the *I* categories describing the rows. The barycenter of a category is the weighted average of the rows in which the weights are the masses rescaled such that the sum of the weights for each category is equal to one. Specifically, the *I* by *J* matrix of barycenters, denoted **R**, is computed as

(2) $$\begin{array}{c} \;R=\mathrm{diag}{\left\{Y^{⊤}M1\right\}}^{-1}Y^{⊤}MX, \end{array}$$

where 1 is an *N* by 1 vector of 1 s and the diagonal matrix diag⁡{**Y** **M**1}^−1^ rescales the masses of the rows such that their sum is equal to one for each category.

### 3.1. Masses and Weights

 The type of preprocessing and the choice of the matrix of masses for the categories (**B**) and the matrix of weights for the variables (**W**) is crucial because these choices determine the type of GPCA used.

For example, discriminant correspondence analysis is used when the data are counts. In this case, the preprocessing is obtained by transforming the rows of **R** into relative frequencies, and by using the relative frequencies of the barycenters as the masses of the rows and the inverse of the column frequencies as the weights of the variables. Another example of GPCA, standard discriminant analysis, is obtained when **W** is equal to the inverse of the within group variance-covariance matrix (which can be computed only when this matrix is full rank). Hellinger distance analysis (also called “spherical factorial analysis”; [32–35]) is obtained by taking the square root of the relative frequencies for the rows of **R** and by using equal weights and masses for the matrices **W** and **M**. Interestingly, the choice of weight matrix **W** is equivalent to defining a generalized Euclidean distance between *J*-dimensional vectors [32]. Specifically, if **x**_*n*_ and **x**_*n*′_ are two *J*-dimensional vectors, the generalized Euclidean squared distance between these two vectors is

(3) $$\begin{array}{c} d_{W}^{2}\left(x_{n},x_{n^{\prime }}\right)={\left(x_{n}-x_{n^{\prime }}\right)}^{⊺}W\left(x_{n}-x_{n^{\prime }}\right). \end{array}$$

### 3.2. GPCA of the Barycenter Matrix

Essentially, BADA boils down to a GPCA of the barycenter matrix **R** under the constraints provided by the matrices **B** (for the *I* categories) and **W** (for the columns). Specifically, the GPCA is implemented by performing a generalized singular value decomposition of matrix **R** [23, 26, 36, 37], which is expressed as

(4) $$\begin{array}{c} R=P\Delta Q^{⊤}\;\text{with}\;P^{⊤}BP=Q^{⊤}WQ=I, \end{array}$$

where Δ is the *L* by *L* diagonal matrix of the singular values (with *L* being the number of nonzero singular values), and **P** (resp., **Q**) being the *I* by *L* (resp., *J* by *L*) matrix of the left (resp., right) generalized singular vectors of **R**.

### 3.3. Factor Scores

The *I* by *L* matrix of factor scores for the categories is obtained as

(5) $$\begin{array}{c} F=P\Delta =RWQ. \end{array}$$

These factor scores are the projections of the categories on the GPCA space and they provide the best separation between the categories because they have the largest possible variance. In order to show this property, recall that the variance of the columns of **F** is given by the square of the corresponding singular values (i.e., the “eigen-value” denoted *λ*) and are stored in the diagonal matrix Λ). This can be shown by combining (4) and (5) to give

(6) $$\begin{array}{c} F^{⊤}BF=\Delta P^{⊤}BP\Delta =\Delta^{2}=\Lambda . \end{array}$$

Because the singular vectors of the SVD are ordered by size, the first factor extracts the largest possible variance, the second factor extracts the largest variance left after the first factor has been extracted, and so forth.

#### 3.3.1. Supplementary Elements

 The *N* rows of matrix **X** can be projected (as “supplementary” or “illustrative” elements) onto the space defined by the factor scores of the barycenters. Note that the matrix **W** **Q** from (5) is a projection matrix. Therefore, the *N* by *L* matrix **H** of the factor scores for the rows of **X** can be computed as

(7) $$\begin{array}{c} H=XWQ. \end{array}$$

These projections are barycentric, which means that the weighted average of the factor scores of the rows of a category gives the factors scores of this category. This property is shown by first computing the barycenters of the row factor scores as (cf. (2)) as

(8) $$\begin{array}{c} \bar{H}=\mathrm{diag}{\left\{YM1\right\}}^{-1}YMH, \end{array}$$

then plugging in (7) and developing. Taking this into account, (5) gives

(9) $$\begin{array}{c} \bar{H}=\mathrm{diag}{\left\{YM1\right\}}^{-1}YMXWQ=RWQ=F. \end{array}$$

### 3.4. Loadings

The loadings describe the variables of the barycentric data matrix and are used to identify the variables important for the separation between the categories. As for standard PCA, there are several ways of defining the loadings. The loadings can be defined as the correlation between the columns of matrix **R** and the factor scores. They can also be defined as the matrix **Q** or as variable “factor scores” which are computed as

(10) $$\begin{array}{c} G=Q\Delta . \end{array}$$

(Note that **Q** and **G** differ only by a normalizing factor).

## 4. Quality of the Prediction

The performance, or quality of the prediction of a discriminant analysis, is assessed by predicting the category membership of the observations and by comparing the predicted with the actual category membership. The pattern of correct and incorrect classifications can be stored in a confusion matrix in which the columns represent the actual categories and in which the rows represent the predicted categories. At the intersection of a row and a column is the number of observations from the column category assigned to the row category.

The performance of the model can be assessed for the observations (e.g., scans or participants) actually used to compute the categories (the set of observations used to generate the model is sometimes called the *training set*). In this case, the performance of the model corresponds to a *fixed* effect model because this assumes that a replication of the experiment would use the same observations (i.e., the same participants and the same stimuli). In order to assess the quality of the model for *new* observations, its performance, however, needs to be evaluated using observations that were not used to generate the model (the set of “new observations” used to evaluate the model is sometimes called the *testing set*). In this case, the performance of the model corresponds to a *random* effect model because this assumes that a replication of the experiment would use the different observations (i.e., different participants and stimuli).

### 4.1. Fixed Effect Model

The observations in the fixed effect model are used to compute the barycenters of the categories. In order to assign an observation to a category, the first step is to compute the distance between this observation and all *I* categories. Then, the observation is assigned to the closest category. Several possible distances can be chosen, but a natural choice is the Euclidean distance computed in the factor space. If we denote by **h**_*n*_ the vector of factor scores for the *n*th observation, and by **f**_*i*_ the vector of factor scores for the *i*th category, then the squared Euclidean distance between the *n*th observation and the *i*th category is computed as

(11) $$\begin{array}{c} d^{2}\left(h_{n},f_{i}\right)={\left(h_{n}-f_{i}\right)}^{⊤}\left(h_{n}-f_{i}\right). \end{array}$$

Obviously, other distances are possible (e.g., Mahalanobis distance), but the Euclidean distance has the advantage of being “directly read” on the map.

#### 4.1.1. Tolerance Intervals

The quality of the category assignment of the actual observations can be displayed using *tolerance* intervals. A tolerance interval encompasses a given proportion of a sample or a population. When displayed in two dimensions, these intervals have the shape of an ellipse and are called *tolerance ellipsoids*. For BADA, a category tolerance ellipsoid is plotted on the category factor score map. This ellipsoid is obtained by fitting an ellipse which includes a given percentage (e.g., 95%) of the observations. Tolerance ellipsoids are centered on their categories. The overlap of the tolerance ellipsoids of two categories reflects the proportion of misclassifications between these two categories for the fixed effect model.

### 4.2. Random Effect Model

The observations of the random effect model are not used to compute the barycenters but are used only to evaluate the quality of the assignment of *new* observations to categories. A convenient variation of this approach is “leave-one-out” (aka jackknife) approach: Each observation is taken out from the dataset and, in turn, is then projected onto the factor space of the remaining observations in order to predict its category membership. For the estimation to be unbiased, the left-out observation should not be used in any way in the analysis. In particular, if the data matrix is preprocessed, the left-out observation should not be used in the preprocessing. So, for example, if the columns of the data matrix are transformed into *Z* scores, the left-out observation should *not* be used to compute the means and standard deviations of the columns of the matrix to be analyzed, but these means and standard deviations will be used to compute the *Z*-score for the left-out observation.

The assignment of a *new* observation to a category follows the same procedure as for an observation from the fixed effect model. The observation is projected onto the original category factor scores and is assigned to the closest category. Specifically, we denote by **X**_−*n*_ the data matrix without the *n*th observation, and by **x**_*n*_ the 1 by *J* row vector representing the *n*th observation. If **X**_−*n*_ is preprocessed (e.g., centered and normalized), the preprocessing parameters will be estimated without **x**_*n*_ (e.g., the mean and standard deviation of **X**_−*n*_ are computed *without* **x**_*n*_) and **x**_*n*_ will be preprocessed with the parameters estimated for **X**_−*n*_ (e.g., **x**_*n*_ will be centered and normalized using the means and standard deviations of the columns of **X**_−*n*_). Then, the matrix of barycenters **R**_−*n*_ is computed and its generalized eigendecomposition is obtained as (cf. (4))

(12) $$\begin{array}{c} R_{-n}=P_{-n}\Delta_{-n}Q_{-n}^{⊤}\;\text{with}\;P_{-n}^{⊤}W_{-n}P_{-n}=Q_{-n}^{⊤}B_{-n}Q_{-n}=I \end{array}$$

(with **B**_−*n*_ and **W**_−*n*_ being the mass and weight matrices for **R**_−*n*_). The matrix of factor scores denoted **F**_−*n*_ is obtained as (cf. (5))

(13) $$\begin{array}{c} F_{-n}=P_{-n}\Delta_{-n}=R_{-n}W_{-n}Q_{-n}. \end{array}$$

The projection of the *n*th observation, considered as the “testing” or “*new* observation,” is denoted ${\tilde{h}}_{n}$ and it is obtained as (cf. (7))

(14) $$\begin{array}{c} {\tilde{h}}_{n}=x_{n}W_{-n}Q_{-n}. \end{array}$$

Distances between this *n*th observation and the *I* categories can be computed from the factor scores (cf. (11)). The observation is then assigned to the closest category. In addition, the jackknife approach can provide an (unbiased) estimate of the position barycenters as well as their standard error (see, e.g., [38], for this approach).

Often in *f* mri experiments, observations are structured in blocks in which observations are not independent of each others (this is the case in most “block designs”). In such cases, a standard leave-one-out approach will overestimate the quality of prediction and should be replaced by a “leave one *block* out” procedure.

#### 4.2.1. Prediction Intervals

In order to display the quality of the prediction for *new* observations, we use *prediction* intervals. Recall that a “leave one out” or jackknife (or “leave one block out”) procedure is used to predict each observation from the other observations. In order to compute prediction intervals, the first step is to project the left-out observations onto the original complete factor space. There are several ways to project a left-out observation onto the factor score space. Here, we propose a two-step procedure. First, the observation is projected onto the factor space of the remaining observations. This provides factor scores for the left-out observation and these factor scores are used to reconstructed the observation from its projection (in general, the left-out observation is imperfectly reconstructed and the difference between the observation and its reconstruction reflects the lack of fit of the model). Then, the reconstructed observation, denoted ${\tilde{x}}_{n}$, is projected onto the full factor score solution. Specifically, a left-out observation is reconstructed from its factor scores as (cf. (4) and (14))

(15) $$\begin{array}{c} {\tilde{x}}_{n}={\tilde{h}}_{n}Q_{-n}^{⊤}. \end{array}$$

The projection of the left-out observation is denoted ${\hat{h}}_{n}$ and is obtained by projecting ${\tilde{x}}_{n}$ as a supplementary element in the original solution. Specifically, ${\hat{h}}_{n}$ is computed as

(16) $$\begin{array}{cc} {\hat{h}}_{n} & ={\tilde{x}}_{n}WQ\;\left(\text{cf}.\;\left(5\right)\right)\\ & ={\tilde{h}}_{n}Q_{-n}^{⊤}WQ\;\left(\text{cf}.\;\left(15\right)\right)\\ & =x_{n}W_{-n}Q_{-n}Q_{-n}^{⊤}WQ\;\left(\text{cf}.\;\left(14\right)\right). \end{array}$$

Prediction ellipsoids are not necessarily centered on their categories (the distance between the center of the ellipse and the category represents the estimation *bias*). Overlap of two predictions intervals directly reflects the proportion of misclassifications for the *new* observations.

## 5. Quality of the Category Separation

### 5.1. Explained Inertia (*R* ^2^) and Permutation Test

In order to evaluate the quality of the discriminant model, we use a coefficient inspired by the coefficient of correlation. Because BADA is a barycentric technique, the total *inertia* (i.e., the “variance”) of the observations to the grand barycenter (i.e., the barycenter of all categories) can be decomposed into two additive quantities: (1) the inertia of the observations relative to the barycenter of their own category, and (2) the inertia of the category barycenters to the grand barycenter. Specifically, if we denote by $\bar{f}$ the vector of the coordinates of the grand barycenter (i.e., each component of this vector is the average of the corresponding components of the barycenters), the total inertia, denoted *ℐ*_Total_, is computed as the sum of the squared distances of the observations to the grand barycenter (cf. (11)):

(17) $$\begin{array}{c} {ℐ}_{\text{Total}}=\overset{N_{}}{\underset{n}{\sum }}m_{n}d^{2}\left(h_{n},\bar{f}\right)=\overset{N_{}}{\underset{n}{\sum }}m_{n}{\left(h_{n}-\bar{f}\right)}^{⊤}\left(h_{n}-\bar{f}\right). \end{array}$$

The inertia of the observations relative to the barycenter of their own category is abbreviated as the “inertia within.” It is denoted *ℐ*_Within_ and computed as

(18) $$\begin{array}{cc} {ℐ}_{\text{Within}} & =\overset{I}{\underset{i}{\sum }}\;\sum_{n\;\text{in}\;\text{category}\;i}m_{n}d^{2}\left(h_{n},f_{i}\right)\\ & =\overset{I}{\underset{i}{\sum }}\;\sum_{n\;\text{in}\;\text{category}\;i}m_{n}{\left(h_{n}-f_{i}\right)}^{⊺}\left(h_{n}-f_{i}\right). \end{array}$$

The inertia of the barycenters to the grand barycenter is abbreviated as the “inertia between.” It is denoted *ℐ*_Between_ and computed as

(19) $$\begin{array}{cc} {ℐ}_{\text{Between}} & =\overset{N_{}}{\underset{n}{\sum }}b_{i}\times d^{2}\left(f_{i},\bar{f}\right)=\overset{I}{\underset{i}{\sum }}b_{i}\times d^{2}\left(f_{i},\bar{f}\right)\\ & =\overset{I}{\underset{i}{\sum }}b_{i}\times {\left(f_{i}-\bar{f}\right)}^{⊤}\left(f_{i}-\bar{f}\right). \end{array}$$

So the additive decomposition of the inertia can be expressed as

(20) $$\begin{array}{c} {ℐ}_{\text{Total}}={ℐ}_{\text{Within}}+{ℐ}_{\text{Between}}. \end{array}$$

This decomposition is similar to the familiar decomposition of the sum of squares in the analysis of variance. This suggests that the intensity of the discriminant model can be tested by the ratio of between inertia by the total inertia, as is done in analysis of variance and regression. This ratio is denoted *R* ^2^ and it is computed as

(21) $$\begin{array}{c} R^{2}=\frac{{ℐ}_{\text{Between}}}{{ℐ}_{\text{Total}}}=\frac{{ℐ}_{\text{Between}}}{{ℐ}_{\text{Between}}+{ℐ}_{\text{Within}}}. \end{array}$$

The *R* ^2^ ratio takes values between 0 and 1, the closer to one, the better the model. The significance of *R* ^2^ can be assessed by permutation tests and confidence intervals can be computed using cross-validation techniques such as the jackknife (see [19]).

### 5.2. Confidence Intervals

The stability of the position of the categories can be displayed using *confidence* intervals. A confidence interval reflects the variability of a population *parameter* or its estimate. In two dimensions, this interval becomes a confidence ellipsoid. The problem of estimating the variability of the position of the categories cannot, in general, be solved analytically and cross-validation techniques need to be used. Specifically, the variability of the position of the categories is estimated by generating *bootstrapped* samples from the sample of observations. A bootstrapped sample is obtained by sampling *with replacement* from the observations. The “bootstrapped barycenters” obtained from these samples are then projected onto the original discriminant factor space and, finally, an ellipse is plotted such that it comprises a given percentage (e.g., 95%) of these bootstrapped barycenters. When the confidence intervals of two categories do not overlap, these two categories are “significantly different” at the corresponding alpha level (e.g., *α* = .05).

It is important to take into account the structure of the design when implementing a bootstrap scheme because the bootstrap provides an unbiased estimate only when the bootstrapped observations are independent [39]. Therefore, when the observations are structured into subtables, these subtables should be bootstrapped in addition to the observations. Conversely, the fixed part of a design should be kept fixed. So, for example, when scans are organized into blocks, the block structure should be considered as fixed [40].

#### 5.2.1. Interpreting Overlapping Multidimensional Confidence Intervals

When a confidence interval involves only one dimension (e.g., when using a confidence interval to compare the mean of two categories), the relationship between hypothesis testing and confidence intervals is straightforward. If two confidence intervals do not overlap, then the null hypothesis is rejected. Conversely, if two confidence intervals overlap, then the null hypothesis cannot be rejected. The same simple relationship holds with a 2-dimensional display as long as all the variance of the data can be described with only two dimensions. If two confidence ellipses (i.e., the 2-dimensional expression of an interval) do not overlap, then the null hypothesis is rejected, whereas if the ellipses overlap, then the null hypothesis cannot be rejected.

In most multivariate analyses (such as BADA), however, the 2-dimensional maps used to display the data represent only *part* of the variance of the data and these displays can potentially be misleading because the position of a confidence ellipse in a 2-dimensional map gives only an approximation of the real position of the intervals in the complete space. Now, if two confidence ellipses do not overlap in at least one display, then the two corresponding categories do not overlap in the whole space (and the null hypothesis can be rejected). However, when two confidence ellipses overlap in a given display, then the two categories may or may not overlap in the whole space (because the overlap may be due to a projection artifact). In this case, the null hypothesis may or may not be rejected depending upon the relative position of the ellipses in the other dimensions of the space. Strictly speaking, when analyzing data laying in a multidimensional space, the interpretations of confidence intervals are correct only when performed in the whole space and the 2-dimensional representations give only an approximation. As a palliative, it is important to examine additional graphs obtained from other dimensions than the first two.

#### 5.2.2. Confidence Intervals: The Multiple Comparison Problem

As mentioned earlier, a confidence interval generalizes a null hypothesis test. And, just like a standard test, the *α*-level chosen is correct only when there are only two categories to be compared because the problem of the inflation of Type I error occurs when there are more than two categories. A possible solution to this problem is to use a Bonferonni or a Šidàk correction (see, [41] for more details). Specifically, for *I* categories, a Bonferonni-corrected confidence interval at the overall (1 − *α*)-level is obtained as

(22) $$\begin{array}{c} 1-\frac{2\alpha }{I\left(I-1\right)}. \end{array}$$

Along the same lines, a Šidàk-corrected confidence level for all pairs of comparisons is expressed as

(23) $$\begin{array}{c} \;{\left(1-\alpha \right)}^{\left(1/2)I(I-1\right)}. \end{array}$$

## 6. Multiple Subject Barycentric Discriminant Analysis (MUSUBADA)

In a multitable analysis, the subtables can be analyzed by projecting the categories and the observations for each subtable. As was the case for the categories, these projections are barycentric because the barycenter of the all the subtables gives the coordinates of the whole table.

### 6.1. Partial Projection

Each subtable can be projected in the common solution. The procedure starts by rewriting (4) as

(24) $$\begin{array}{c} R=P\Delta Q^{⊤}=P\Delta {\left[Q_{1},\ldots ,Q_{k},\ldots ,Q_{K}\right]}^{⊤}, \end{array}$$

where **Q**_*k*_ is the *k*th subtable (comprising the *J*_*k*_ columns of **Q** corresponding to the *J*_*k*_ columns of the *k*th block). Then, to get the projection for the *k*th subtable, (5) is rewritten as

(25) $$\begin{array}{c} F_{k}=KX_{k}W_{k}Q_{k}, \end{array}$$

where **W**_*k*_ is the weight matrix for the *J*_*k*_ columns of the *k*th block.

Equation (25) can also be used to project supplementary rows corresponding to a specific subtable. Specifically, if **x**_sup⁡,*k*_^⊺^ is a *J*_*k*_ row vector of a supplementary element to be projected according to the *k*th subtable, its factor scores are computed as (**x**_sup⁡,*k*_ is supposed to have been preprocessed as **X**_*k*_)

(26) $$\begin{array}{c} f_{\sup,k}=Kx_{\sup,k}W_{k}Q_{k}. \end{array}$$

### 6.2. Inertia of a Subtable

Recall from (6) that, for a given dimension, the variance of the factor scores of all the *J* columns of matrix **R** is equal to the eigenvalue of this dimension. Each subtable comprises a set of columns, and the contribution of a subtable to a dimension is defined as the sum of this dimension squared factor scores of the columns comprising this subtable. Precisely, the inertia for the *k*th table and the *ℓ*th dimension is computed as

(27) $$\begin{array}{c} {ℐ}_{\ell ,k}=\sum_{j\in Jk}w_{j}f_{\ell ,j}^{2}. \end{array}$$

Note that the sum of the inertia of the blocks gives back the total inertia:

(28) $$\begin{array}{c} \lambda_{\ell }=\sum_{k}{ℐ}_{\ell ,k}. \end{array}$$

#### 6.2.1. Finding the Important Subtables

The subtables that contribute to the discrimination between the classes are identified from their partial contributions to the inertia (see (27)).

### 6.3. Subtable Normalization and Relationship with Other Approaches

In the current example, the subtables are simply considered as sets of variables. Therefore, the influence of a given subtable reflects not only its number of variables, but also its factorial structure because, everything else being equal, a subtable with a large first eigenvalue will have a large influence on the first factor of the whole table. By contrast, a subtable with a small first eigenvalue will have a small effect on the first factor of the whole table. In order to eliminate such discrepancies, the *multiple factor analysis* approach [15, 17, 36] normalizes each subtable by dividing each element of a subtable by its first singular value.

An alternative normalization can be derived from the STATIS approach [16, 18, 19, 42, 43]. In this framework, each subtable is normalized from an analysis of the *K* by *K* matrix of the between-subtable *R*_*V*_ matrix (recall that the *R*_*V*_ coefficient plays a role analogous to the coefficient of correlation for semipositive definite matrices, see [41, 44, 45]). In this framework, subtables are normalized in order to reflect how much information they share with the other subtables.

In the context of MUSUBADA, these normalization schemes can be performed on the subtables of the matrix **X**, but their effects are easier to analyze if it is performed on the barycentric matrix **R**. These two normalizing schemes can also be combined with the subtables being first normalized according the multiple factor analysis approach and then using the STATIS approach.

### 6.4. Programs

MATLAB and R programs are available from the home page of the first author (at http://www.utdallas.edu/~herve/).

### 6.5. MUSUBADA and Other Classifiers

 MUSUBADA is a linear classifier that can handle multiple participants when these participants are represented by different numbers of voxels or ROIs. As such, its performance is likely to be closely related to the performance of other linear classifiers (see [46] for a general presentation in the context of MVPA models) such as (linear) support vector machines (SVM, see, e.g., [47] for an application of SVM to *f* mri data) or linear discriminant analysis (in general performed on the components of a PCA, see [48]). When the structure of the data (i.e., spatial normalization) allows the use of different linear techniques, these techniques will likely perform similarly (numerical simulations performed with some examples confirm this conjecture). However, as indicated previously, most of these techniques will not be able to handle the integration of participants with different number of voxels or ROIs. Canonical STATIS (CANOSTATIS [49]) can integrate discriminant analysis problems obtained with a different number of variables, but in its current implementation, it requires that the data of each participant be full rank, a restriction that makes difficult for this technique to be used with large brain imaging datasets. It has been suggested [19] to use approaches such as ridge or regularization techniques (see, e.g., [50], for a review) to make CANOSTATIS able to handle multicolinearity, and such a development could be of interest for brain imaging. However, CANOSTATIS works on Mahanalobis distance matrices between categories and does not have—in its current implementation—ways of predicting membership of scans to categories and cannot identify the voxels important for the discrimination between categories.

The closest statistical technique to MUSUBADA is probably mean centered partial least-square correlation (MC-PLSC, see [51–53], for presentations and reviews), which is widely used in brain imaging. In fact, when the data are spatially normalized, MUSUBADA and MC-PLSC will analyze (i.e., compute the singular value decomposition of) the same matrix of barycenters. MUSUBADA adds to MC-PLSC the possibility of handling different numbers of voxels per participant, ROI analysis, and also an explicit prediction of group categories which is lacking from the standard implementation of MC-PLSC. So, MUSUBADA can be seen as a generalization of MC-PLSC that can handle multiple ROIs, different numbers of voxels per participant, and can predict category membership.

## 7. An Example

As an illustration, we are using the dataset originally collected by Connolly et al. [54]. In this experiment, 10 participants performed a one-back recognition memory task on visual stimuli from seven different categories: female faces, male faces, monkey faces, dog faces, houses, chairs, and shoes. The stimuli were presented in 8 runs of 7 blocks—one block per category per run. A block consisted of 16 image presentations from a given category with each image presented for a duration of 500 ms separated by a 2 s interstimulus interval (2.5 s stimulus onset asynchrony) for a total block duration of 40 s. Full brain scans were acquired every 2 s (TR = 2000 ms). Blocks were separated by 12 s intervals of fixation. A different random ordering of category blocks was assigned to each run, and each subject saw the same set of runs (i.e., the same set of random category block orderings), however, each subject saw the runs in a different randomly assigned run order. For analysis, the runs for each subject were reordered to a canonical run order so that a single set of time-point labels could be used for all subjects. Time-point labels were coded such that the first four TRs of each block were discarded so that only the maximal evoked BOLD response was coded for each category block. The time-course for each run was thus coded with 16 consecutive full-brain scans (covering 32 sec starting 8 secs after the onset of the block) assigned to each stimulus block. No regression analyses were performed to estimate the average BOLD response for the stimulus categories; rather, each brain image (16 per stimulus block) contributed to a unique row in the data matrix.

### 7.1. Data Acquisition

 BOLD *f* mri images were acquired with gradient echoplanar imaging using a Siemens Allegra head only 3T scanner. The *f* mri images consisted of thirty-two 3 mm thick axial images (TR = 2000, TE = 30 ms, flip angle = 90, 64 × 64 matrix, FOV = 192 × 192 mm) and included all of the occipital and temporal lobes and the dorsal parts of the frontal and parietal lobes. High-resolution T1-weighted structural images were also acquired.

### 7.2. Imaging Preprocessing

 Preprocessing of the *f* mri data included slice timing correction, volume registration to correct for minor head movements, correction of extreme values, and mean correction for each run. No spatial normalization or coregistration was performed.

### 7.3. Region of Interest

 For each participant, a mask was hand drawn based on anatomical landmarks obtained from the structural scan. The mask was used to identify one ROI the Ventrotemporal (VT) area ROI which included the inferior temporal, fusiform, and parahippocampal gyri. Because the ROI was anatomically defined, its size (in number of voxels) differed from participant to participant (from 2791 to 4815). The total number of voxels (for all 10 participants) was equal to 39,163.

### 7.4. Statistical Preprocessing

 Prior to the analysis, the data were centered by removing the average scan. In addition, a subtable normalization was performed on each participant. For each participant, a singular value decomposition (SVD) was performed and all the voxels of a given participant were divided by the first singular value obtained from this SVD. In addition, each row of the data table was normalized such that the sum of the squares of its elements was equal to one.

### 7.5. Final Data Matrices

**Figure 2:**
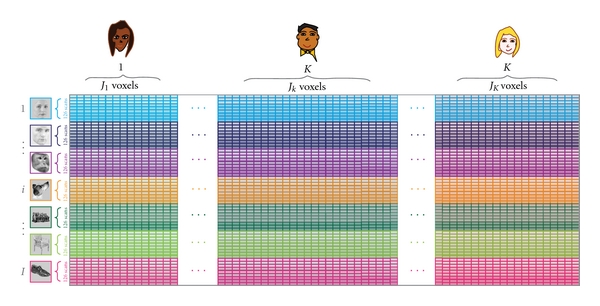
Design and data matrix for the MUSUBADA example. A row represents one image belonging to one of seven categories of images (female faces, male faces, monkey faces, dog faces, houses, chairs, and shoes). A column represents one voxel belonging to one of the ten participants. At the intersection of a row and a column we find the value of the activation of one voxel of one participant (i.e., column) when this participant was watching a given image (i.e., row) Note that the participants' ROI was drawn anatomically for each participant, so the number of voxels differs between participants (i.e., the scans are *not* morphed into a standardized space).

The data matrix, **X**, is a 896 × 39,163 matrix. The rows of **X** correspond to the 896 scans which were organized in 8 blocks of 7 stimulus categories comprising 16 brain images each. Therefore, each of the seven stimulus groups was represented by 16 × 8 = 128 brain images. The 39,163 columns of **X** represented the voxel activations of all the participants and are organized in 10 subtables (one per participant; the data could be fitted in such a simple format because both the scan block orders were constant for all participants). The design matrix **Y** was a 896 × 7 matrix coding for the categories. A schematic of the data matrix is shown in Figure 2.

### 7.6. Goal: Finding the Categories

**Figure 3:**
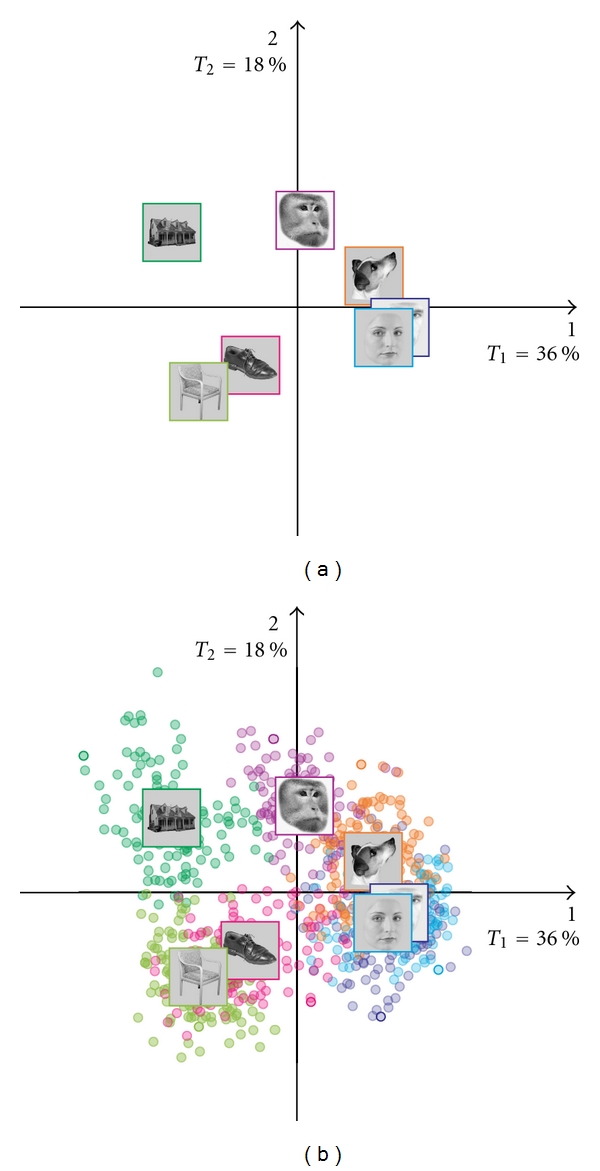
BADA on the scans: Category barycenters. (a) Barycenters, and (b) Barycenters with observations. Dimension 1 (on the horizontal) separates the faces from the objects. Dimension 2 (on the vertical) separates the large and the small objects.

**Figure 4:**
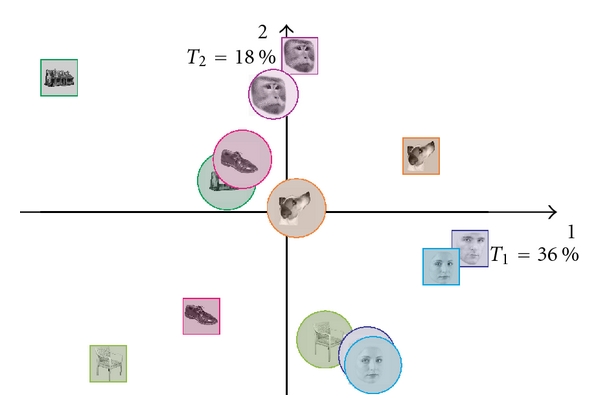
BADA on the pictures used as stimuli projected as supplementary elements on the solution obtained from the *f* mri scans. Picture categories are shown in circles and *f* mri groups in squares. The picture category line on up the second dimension but not on the first.

The goal of the analysis was to find out if it was possible to discriminate between scans coming from the different categories of images. To do this, each category in **X** was summed to create the 7 × 39,163 barycentric matrix **R** (see (2)). We then computed the BADA on the **R** matrix which provided the map shown in Figure 3. The first dimension of this map shows a clear opposition of faces (i.e., female, male, and dog faces) and objects (i.e., houses, chairs, and shoes). This dimension can be interpreted as a *semantic* dimension: faces versus nonfaces. The second dimension, in contrast, separates the smaller from the larger objects (i.e., chairs and shoes versus houses) and also reflects variation among the faces, as it separates monkey from human faces (with dog faces in-between). It is also possible that dimension 2 reflects a combination of features (e.g., low level visual features). In order to support this interpretation, we ran a BADA on the stimuli (see, e.g., [55] for a similar idea) and obtained a set of factor scores for the picture groups. We then projected the picture categories, as supplementary elements, onto the factor space obtained from the *f* mri data. We used the procedure described in Abdi [56] after rescaling the picture factor scores so that the factor scores for the first dimension have the same variance (i.e., eigenvalue) as the first factor scores of the analysis performed on the participants. The results are shown in Figure 4. In agreement with our interpretation, we found that the categories obtained from the pictures lined up with dimension 2 and had very little variance on dimension 1.

### 7.7. Stability of the Discriminant Model

#### 7.7.1. Fixed Effect Model

 To examine the reliability of the fixed effect model, we looked at the fixed effect confusion matrix and the tolerance intervals. The fixed effect confusion matrix is shown in Table 1. We see that, for the fixed effect model, classification was very good: the model correctly classified 126/128 female faces, 127/128 male faces, all 128 monkey faces, 127/128 houses, all 128 chairs, 124/128 shoes, and all 128 dog faces.

**Figure 5:**
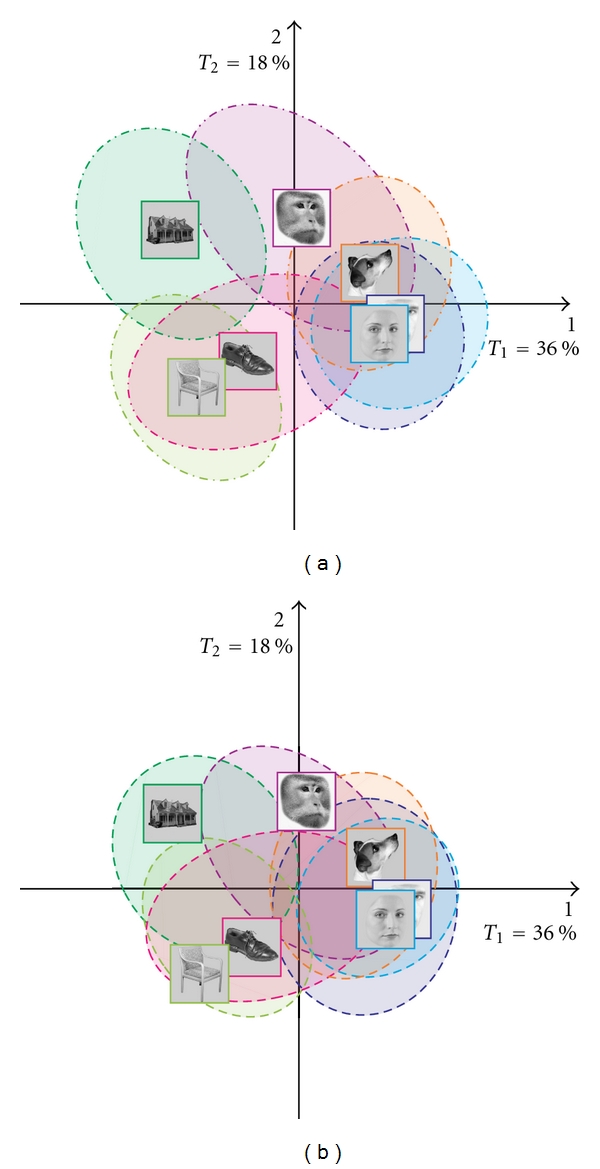
BADA on the scans: (a) tolerance ellipses, and (b) prediction ellipses.

To answer the question of separability, we looked at the tolerance intervals (shown in Figure 5(a)). The tolerance intervals include 95% of the projections of the observations centered around their respective categories. Recall that tolerance intervals reflect the accuracy of the assignment of scans to categories. Therefore, when two categories do not overlap for at least one dimension, they can be considered as separable (note, that, again *all* dimensions need to be considered and that a two-dimensional display is accurate only when all the variance of the projections is two dimensional). We show here the tolerance intervals only for dimensions 1 and 2, but we based our interpretation on the whole space.

Large differences were found between houses and female faces, between houses and male faces, between houses and dog faces, between chairs and female faces, between chairs and male faces, between chairs and monkey faces, and between chairs and dog faces. The variance of the projection of the individual scans (as represented by the area of the ellipsoids) varies with the categories with monkey faces and shoes displaying the largest variance and with human faces showing the smallest variance.

Interestingly, the female and male faces are close to each other and overlap on most dimensions, this pattern suggests that the scans from these categories were very similar.

#### 7.7.2. Random Effect Model

The random effect model evaluates the performance of the model for *new* observations. The current experiment used a block design, and the scans within a block are generally not independent because of the large time correlation typical of *f* mri experiments, whereas scans between blocks can be considered independent (because of the resting interval between blocks). Therefore, in order to compute the random effect confusion matrix, we used a “leave-one-block-out” procedure in which we left the *whole block* out in order to avoid confounding prediction with time correlation (see [38]). This resulted in the confusion matrix shown in Table 2. As expected, classification for the random effect model was not as good as that for the fixed effect model, with 47/128 female faces, 34/128 male faces, 53/128 monkey faces, 81/128 houses, 96/128 chairs, 45/128 shoes, and 53/128 dog faces correctly classified. So, overall the categories are well separated in the random effect model. However, the human females and male faces were not separated and seem to constitute a single category (with female faces showing more variability the male faces).

Like the fixed effect model, we can examine the stability of the random effect model. The prediction intervals include 95% of the bootstrap sample projections. Note that prediction intervals may not be centered around the category mean, because *new* observations are unlikely to have the exact *same* mean as the *old* observations. Recall that prediction intervals reflect the accuracy of assignment of *new* scans to categories and represent where observations would fall in the population. The prediction intervals for the example are shown in Figure 5(b). Note that the large overlap of the categories ellipsoids suggests that some scans of a given categories can be misclassified for scans from any other category.

### 7.8. Quality of Category Separation

#### 7.8.1. *R* ^2^ and the Permutation Test

The quality of the model was also evaluated by computing the percentage of variance explained by the model. This gave a value of *R* ^2^ = .72 with a probability of *P* < .00001 (by permutation test) which confirms the quality of the model.

#### 7.8.2. Confidence Intervals

**Figure 6:**
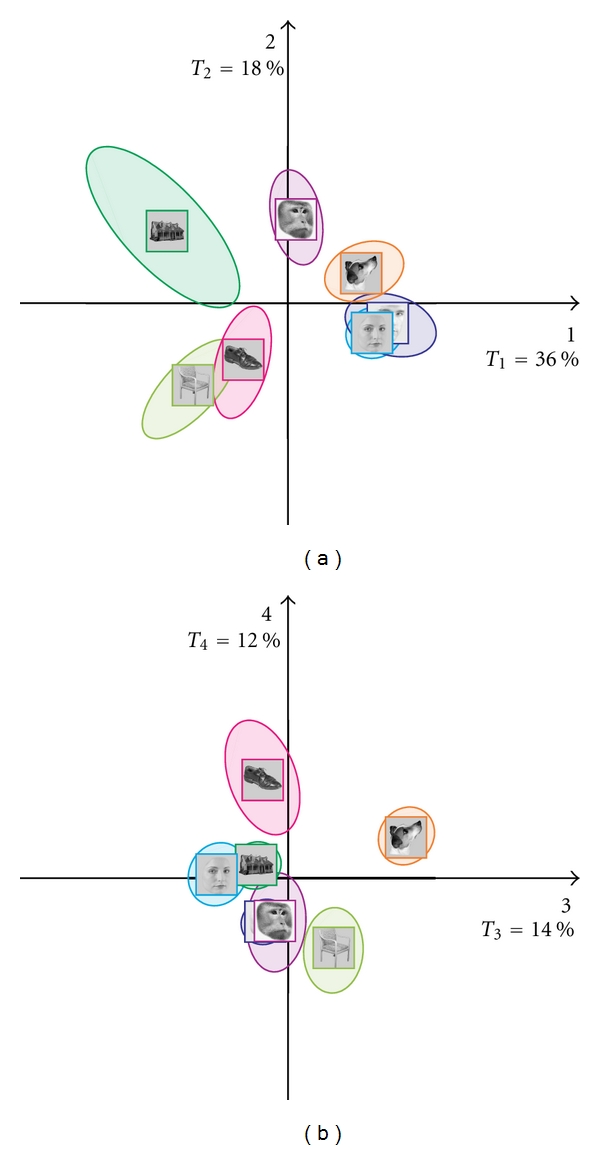
BADA on the scans confidence ellipses: (a) dimensions 1 and 2, and (b) dimensions 3 and 4.

To test whether category discrimination was significant, we used 95% confidence intervals.  Figure 6shows the 95% confidence ellipses for each category on the maps made from Dimensions 1-2 and 3-4. The configuration of the ellipses indicates that all categories are reliably separated, but the separation of some categories is stronger than some others. Specifically, the male and female faces are separated only on the 3-4 map and their distance is relatively small, and this indicates that their separation—even if significant—is small.

### 7.9. Subtable Projections

#### 7.9.1. Partial Inertias of the Participants

**Figure 7:**
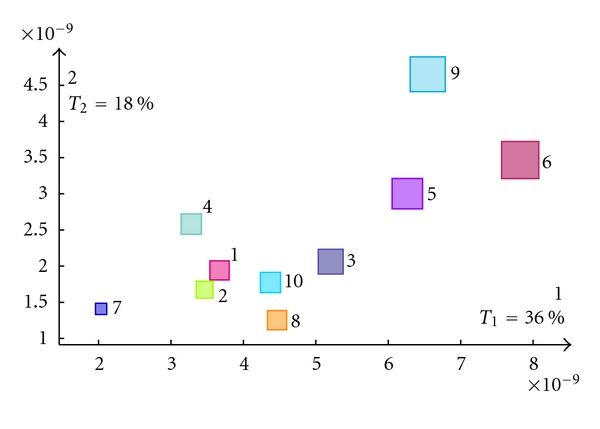
Partial inertias of the participants subtables. The size of a square is proportional to the overall contribution of the participant.

The respective importance of the participants is expressed by the partial inertia (cf. (27)) of their groups of voxels (i.e., the subtable associated to each subject). The partial inertias are plotted in Figure 7. We can see that there is some difference between the participants in the way they express the effect. For example, participant 7 shows the least overall separation between categories, participant 9 shows the clearest overall separation between categories.

#### 7.9.2. Projecting the Subtables as Supplementary Elements

**Figure 8:**
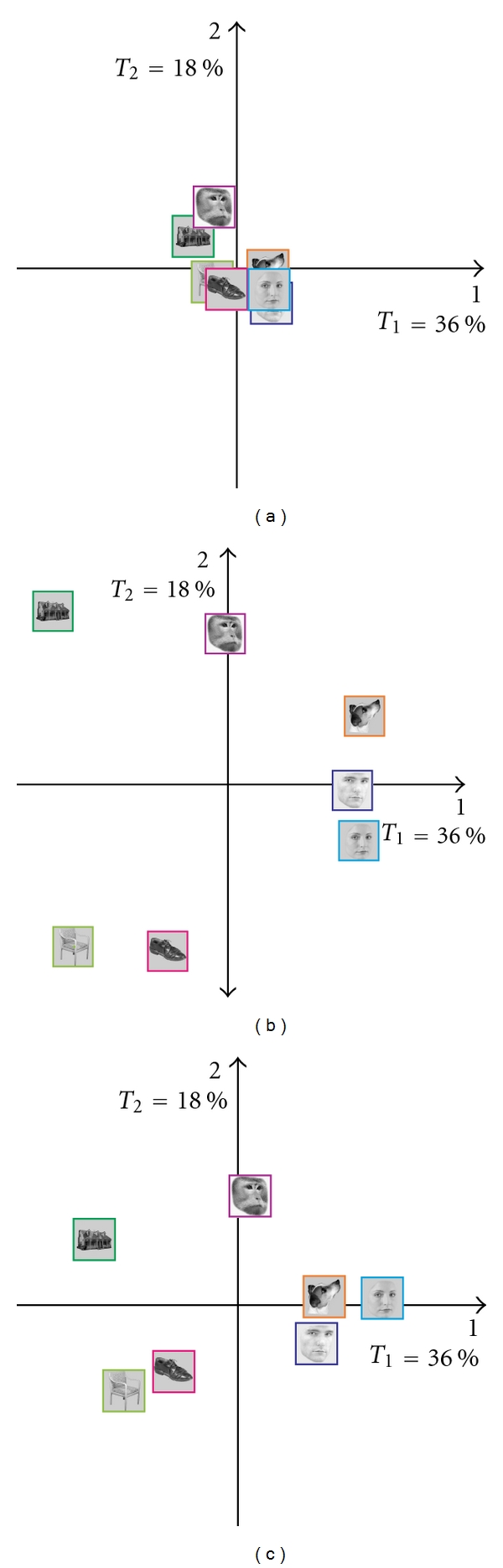
Three interesting participants (a) participant 7, (b) participant 9, and (c) participant 6 (cf. Figure 7). All participants show the same overall configuration, but some participants display a clearer effect.

Interestingly, the overall factorial configuration is very similar for all participants, but some participants exhibit clearer configurations. In order to illustrate this point, in Figure 8, we show the map of participant 7 who shows the least overall separation between categories, participant 9 who shows the clearest overall separation between categories, and participant 6 who shows the clearest separation between categories on dimension 1 (see Figure 7).

## 8. Conclusion

 Multiple subject barycentric discriminant analysis is particularly well suited for the analysis of neuroimaging data because it does not require brains to be spatially normalized. In addition, MUSUBADA can handle very large data sets (with more variables than observations). Even though we did not illustrate this property here, with MUSUBADA, we can also plot (i.e., with “glass brains”) the voxels important for discriminating between categories. Therefore, MUSUBADA can be used to study the similarity structure of the categories as well as that of the voxels. In addition, MUSUBADA could accommodate more sophisticated designs than the one illustrated here. For example, MUSUBADA could analyze design for which several ROIs are defined per participant (see, e.g., [57]), or different categories of participants (e.g., old versus young participants as in [58]). Finally, because MUSUBADA incorporates inferential components, it complements other popular approaches such as partial least squares [53, 59] which are widely used for brain imaging data.

## Figures and Tables

**Table 1:** Fixed effect confusion matrix.

Assigned group	Actual group						
	Female face	Male face	Monkey	House	Chair	Shoe	Dog
Female face	*126*	1	0	0	0	0	0
Male face	0	*127*	0	0	0	0	0
Monkey	0	0	*128*	0	0	0	0
House	0	0	0	*127*	0	0	0
Chair	0	0	0	0	*128*	0	0
Shoe	0	0	0	1	0	*124*	0
Dog	2	0	0	0	0	4	*128*

**Table 2:** Random effect confusion matrix.

Assigned group	Actual group						
	Female face	Male face	Monkey	House	Chair	Shoe	Dog
Female face	*35*	59	14	1	1	3	35
Male face	52	*40*	15	0	0	8	8
Monkey	11	6	*63*	13	5	6	23
House	0	0	14	*90*	14	6	0
Chair	3	1	2	6	*80*	27	1
Shoe	5	5	2	12	28	*55*	11
Dog	22	17	18	6	0	23	*50*
